# Morphology of Polymer Brushes in the Presence of Attractive Nanoparticles: Effects of Temperature

**DOI:** 10.3390/ijms24010832

**Published:** 2023-01-03

**Authors:** Afshin Eskandari Nasrabad, Rozita Laghaei, Rob D. Coalson

**Affiliations:** 1 Department of Computational and Systems Biology, University of Pittsburgh, Pittsburgh, PA 15260, USA; 2 Pittsburgh Supercomputing Center, Carnegie Mellon University, 300 South Craig St., Pittsburgh, PA 15213, USA; 3 Department of Chemistry, University of Pittsburgh, Pittsburgh, PA 15260, USA

**Keywords:** polymer brush, responsive materials, computer simulation, molecular dynamics simulation, nanoparticle, nanodevice, nanovalve

## Abstract

We study the role of temperature on the structure of pure polymer brushes and their mixture with attractive nanoparticles in flat and cylindrical geometries. It has previously been established that the addition of such nanoparticles causes the polymer brush to collapse and the intensity of the collapse depends on the attraction strength, the nanoparticle diameter, and the grafting density. In this work, we carry out molecular dynamics simulation under good solvent conditions to show how the collapse transition is affected by the temperature, for both plane grafted and inside-cylinder grafted brushes. We first examine the pure brush morphology and verify that the brush height is insensitive to temperature changes in both planar and cylindrical geometries, as expected for a polymer brush in a good solvent. On the other hand, for both system geometries, the brush structure in the presence of attractive nanoparticles is quite responsive to temperature changes. Generally speaking, for a given nanoparticle concentration, increasing the temperature causes the brush height to increase. A brush which contracts when nanoparticles are added eventually swells beyond its pure brush height as the system temperature is increased. The combination of two easily controlled external parameters, namely, concentration of nanoparticles in solution and temperature, allows for sensitive and reversible adjustment of the polymer brush height, a feature which could be exploited in designing smart polymer devices.

## 1. Introduction

Recent work has demonstrated that the morphology of polymer brushes can be controlled by introducing into solution nanoparticles that attract to monomers in the brush [1,2,3,4,5]. These attractions provide an enthalpic driving force that encourages nanoparticles to partition into the polymer brush. This, in turn, leads to compression or expansion of the brush, depending on conditions such as the brush grafting density, nanoparticle-monomer binding strength, and the concentration of nanoparticles in solution. For small nanoparticles (on the order of the monomer size), a sharp, pronounced collapse transition can occur as the concentration of nanoparticles is incrementally increased. The extent and sharpness of the transition increases with the nanoparticle-monomer interaction strength.

Nanoparticles (molecules much larger than H_2_O solvent molecules) have been experimentally shown in several instances to infiltrate into a polymer brush and in doing so alter the brush extension. Many of these systems have been inspired by the biological Nuclear Pore Complex [6,7,8], in which the pore is lined with natively unfolded protein filaments that attach to the pore scaffold and form a mesh which is spontaneously infiltrated by specific globular proteins denoted as Nuclear Transport Factors (NTFs). A key factor in the spontaneous partitioning of these NTFs into the NPC pore mesh is attractive hydrophobic interactions (hydrophobic contacts) between parts of the NTFs and hydrophobic amino acid segments in the pore lining nucleoporin proteins [9,10,11]. Several mimetic systems have been synthesized in which protein filaments extracted from the NPC are grafted to a planar solid surface at a density sufficient that the protein filaments form a polymer brush. The brush is then exposed to NTFs that are added to the solution. These NTFs (the sticky “nanoparticles”) are observed to infiltrate into the brush and contract or expand its shape [12,13,14,15]. Following up on these observations, Amemiya et al. [16] identified hydrophobic anionic organic molecules which functioned similarly to NTF’s in that they partitioned into the nucleoporin mesh in the biological NPC, and caused contraction of the mesh near the NPC scaffold. Subsequently, opening and closing of a PEG brush-coated synthetic nanopore using a specially designed antibody molecule (the appropriate nanoparticle in this system) was demonstrated [17]. Thus, a range of possibilities for partitioning attractive nanoparticles into a polymer brush with concomitant modification of the brush morphology have been demonstrated using both biological and synthetic ingredients.

To date, many studies of polymer brush/nanoparticle composite systems have focused on changes that occur at a fixed temperature [13,15,18,19,20,21]. Herein we explore the effect of temperature variation on nanoparticle infused brushes. In general, we would expect that decreasing temperature encourages infiltration of the brush by nanoparticles, concomitant with compression of the brush, which increases the number of contacts that each nanoparticle can have with polymer beads. Qualitatively, decreasing temperature at constant nanoparticle-monomer attraction should produce the same trends as increasing the nanoparticle-monomer attraction at a fixed temperature. A more quantitative connection between nanoparticle-monomer interaction strength and temperature can be anticipated by analyzing the Boltzmann factor that arises from the chosen interaction potential, as will be outlined below.

In order to better understand these phenomena, we have performed coarse-grained Molecular Dynamics (MD) simulations of a system consisting of nanoparticles that interact with a polymer brush over a range of temperatures in both plane-grafted and inside cylinder-grafted brush geometries. We find that in the presence of attractive nanoparticles, the brush conformation becomes very sensitive to the system temperature. Under appropriate nanoparticle concentrations, the brush can be made to significantly contract or to expand by varying the temperature appropriately. This extends the degree of experimental control over the morphology. There are two easily adjusted (and reversible) control parameters, namely, the nanoparticle concentration and the system temperature, which can be adjusted independently.

The outline of the paper is as follows. In Section 2, we describe the coarse-grained MD simulation systems, considering both plane-grafted and cylindrically grafted polymer brushes. We present results of these MD simulations, including the dependence of both monomer and nanoparticle distributions on temperature. A variety of temperatures and nanoparticle-monomer coupling strengths are explored. Section 3 contains Discussion and Conclusions. In Section 4 we explain the methods and materials.

## 2. Results

### 2.1. Pure Brush

The structure of polymer chains grafted to flat and curved surfaces has been the subject of many theoretical as well as computer simulation studies [22,23,24,25,26,27,28,29,30,31,32,33,34,35,36]. The grafting density and the degree of polymerization (the number of monomers in a single polymer chain) are two key parameters that characterize a simple flat polymer brush system, and most studies have focused on the effects of these two variables on the brush structure. In this section, we present results of MD simulations for a pure brush, focusing on the effects of temperature variation. Details of the simulation procedures are presented in Methods and Materials (Section 4).

**Figure 1:**
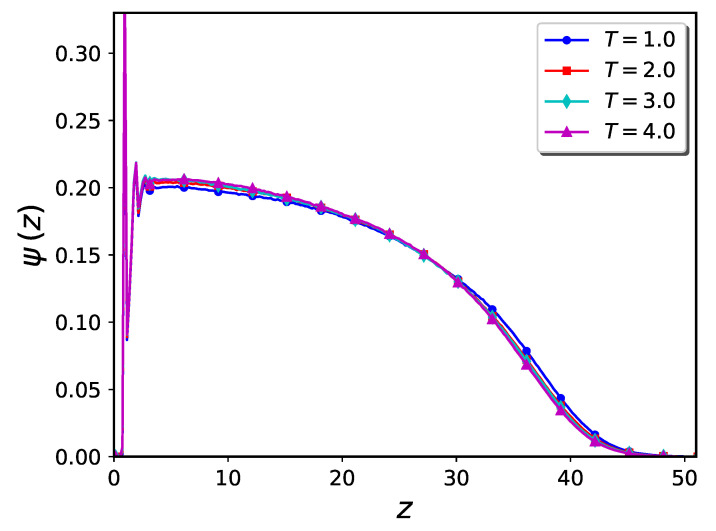
Monomer density profile of pure brushes at different temperatures in flat geometry. Each polymer chain consists of 100 linked beads and the grafting density is $\sigma_{g}=0.0625$ (corresponding to an average grafting distance of 4.0).

MD simulation results for the monomer density profile of a pure brush in the flat system with the grafting density $\sigma_{g}=0.0625$ (corresponding to an average grafting distance of 4.0) are shown in Figure 1 at four temperatures: T= 1, 2, 3, and 4 (in reduced units $\epsilon /k_{B}$, where $k_{B}$ is Boltzmann’s constant). It can be seen from the figure that the system temperature has only a small effect on the density profile. This behavior is expected since the polymer chains are immersed in a good solvent, corresponding to the fact that only excluded volume repulsion is included in the monomer-monomer pair potential $U_{mm}$. In such conditions, as discussed in detail below, the polymer morphology is not sensitive to temperature changes and hence the system is said to be athermal.

**Figure 2:**
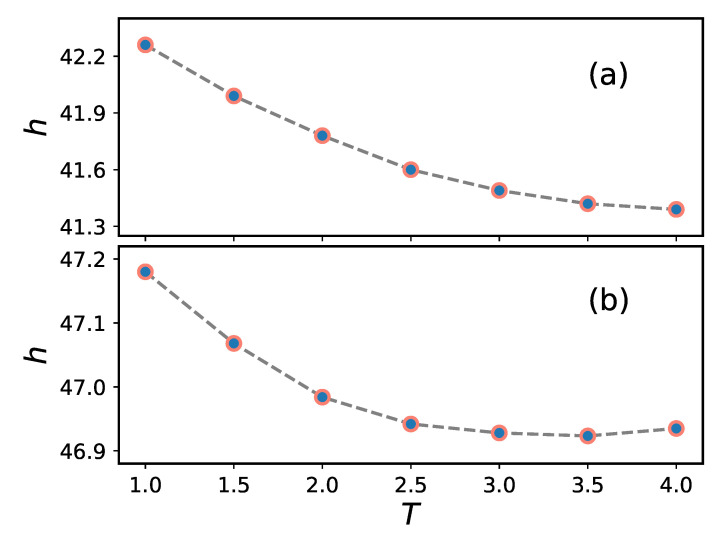
Average height of the polymer brushes considered in Figure 1 as a function of temperature in flat (**a**) and cylindrical (**b**) geometries. (The projection method is used to calculate brush heights in the cylindrical geometry).

Results for the corresponding brush heights are shown in Figure 2 for both flat and cylindrical geometries. (Calculation of properties for the cylindrical brush system are discussed in detail below.) Upon increasing the temperature, the brush height decreases slowly. A rise in temperature from T=1 to 4 causes the brush height to decrease by 0.87 and 0.24 in the flat and cylindrical systems, respectively, out of total brush heights of ca. 42 and 47, respectively.

### 2.2. Brush plus Nanoparticles—Flat Geometry

Changes in morphology of a polymer brush due to the inclusion of nanoparticles in flat and cylindrical geometries have been investigated previously [1,2,3,4,5,13,37]. From these studies, we know that adding attractive nanoparticles to the system typically causes the brush to contract. This trend continues until the brush height reaches a minimum value. Adding more nanoparticles then gives rise to a swollen brush, causing the brush height to increase. In this section we present MD simulations of the brush morphology in the presence of attractive nanoparticles, with an emphasis on the influence of system temperature.

**Figure 3:**
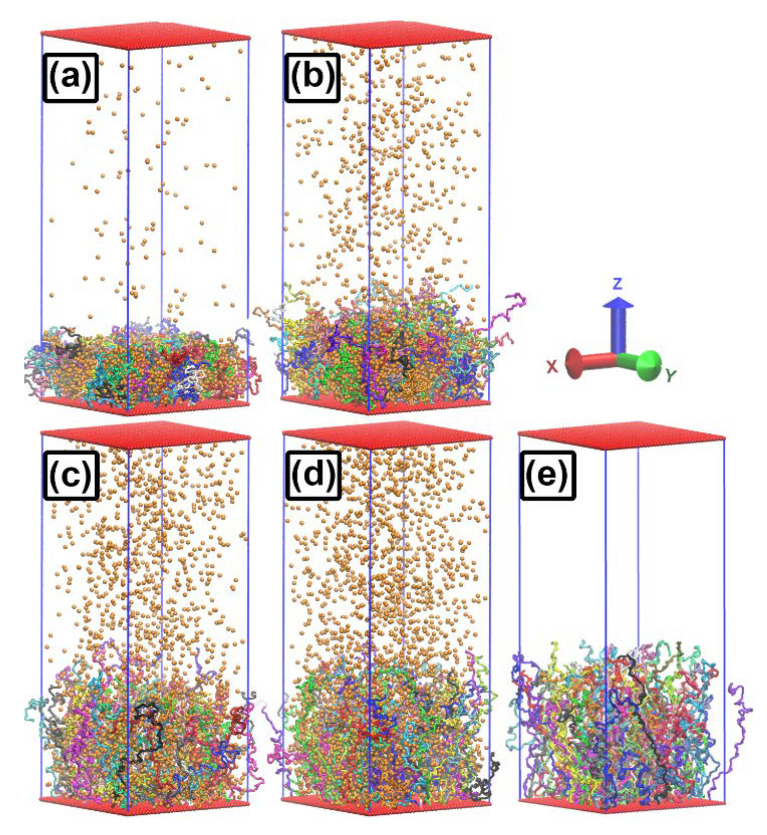
Snapshots of MD simulation cells for planar brush. Results are shown for $\epsilon_{b}=1.5$ and temperatures T=1.0 (**a**), 1.25 (**b**), 1.5 (**c**), and 1.75 (**d**). Simulation setup consists of 100 polymer grafted chains together with 3000 nanoparticles. Panel (**e**) shows an MD snapshot from a pure brush (no nanoparticle) simulation run at T=1.0, included for comparison to systems (**a**–**d**).

We carried out computer simulations in the presence of attractive nanoparticles with two different nanoparticle sizes, $\sigma_{n}=1$ and 3. For the smaller nanoparticle size, we considered two binding strengths, $\epsilon_{b}=1.0$ and 1.5, and for the larger one we considered only $\epsilon_{b}=1.25$. Figure 3 shows the simulation boxes containing 100 grafted polymer chains (each consisting of 100 monomer beads) plus 3000 nanoparticles with $\sigma_{n}=1$ and $\epsilon_{b}=1.5$ at four temperatures T=1.0 (a), 1.25 (b), 1.5 (c), and 1.75 (d). Evidently, increasing the temperature causes both the brush height and the concentration of nanoparticles in the solution to increase. In panel (e) of this figure a snapshot of the corresponding pure brush (nanoparticle free) system at temperature T=1.0 is presented for comparison. As shown in the previous subsection, the brush morphology varies little with temperature in the absence of nanoparticles. Thus, it is apparent that having attractive nanoparticles in the system is required for the brush height to change significantly when the system temperature is altered.

**Figure 4:**
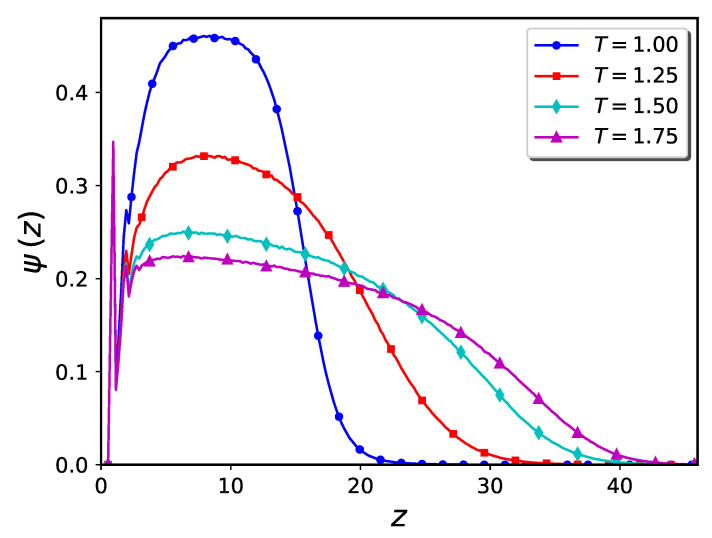
Monomer density profile of brushes at different temperatures corresponding to the snapshots shown in Figure 3a–d.

**Figure 5:**
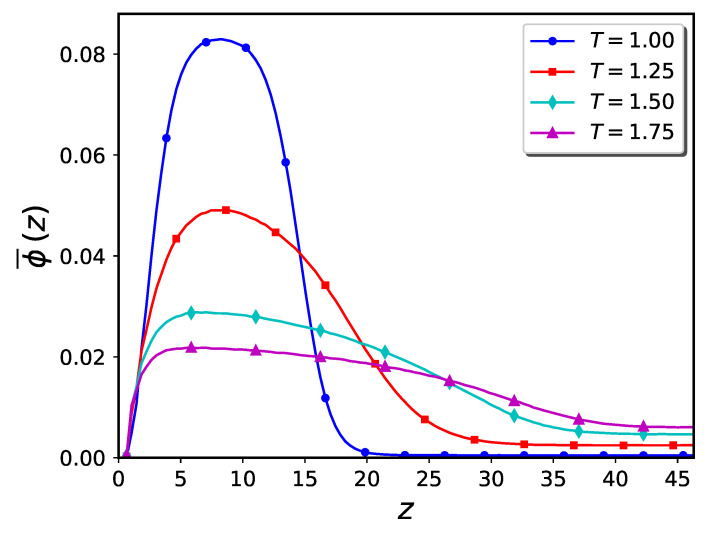
Density profile of nanoparticles at different temperatures corresponding to the snapshots shown in Figure 3a–d.

The corresponding density profiles of the polymer brushes and the nanoparticles are shown in Figure 4 and Figure 5, respectively. The nanoparticle density is notated as $\bar{\phi }$, where the overbar indicates that the nanoparticle density function is normalized to unity, i.e., $\int_{0}^{L_{z}}\bar{\phi }\left(z\right)dz=1$. Upon raising the temperature, the concentration of polymer beads and nanoparticles diminishes in the region close to the substrate, resulting in an increase in the brush height.

**Figure 6:**
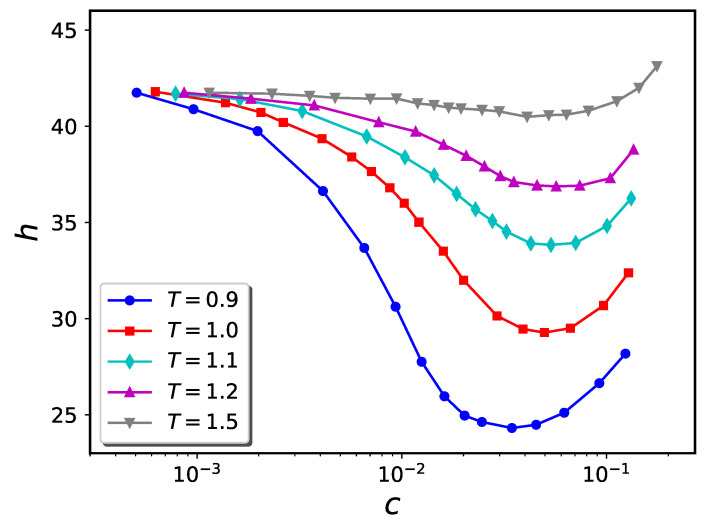
Temperature dependence of h−c diagram for systems with nanoparticle diameter $\sigma_{n}=1$ and $\epsilon_{b}=1.0$ in flat geometry. The brush height of the pure brush is ca. 42.

**Figure 7:**
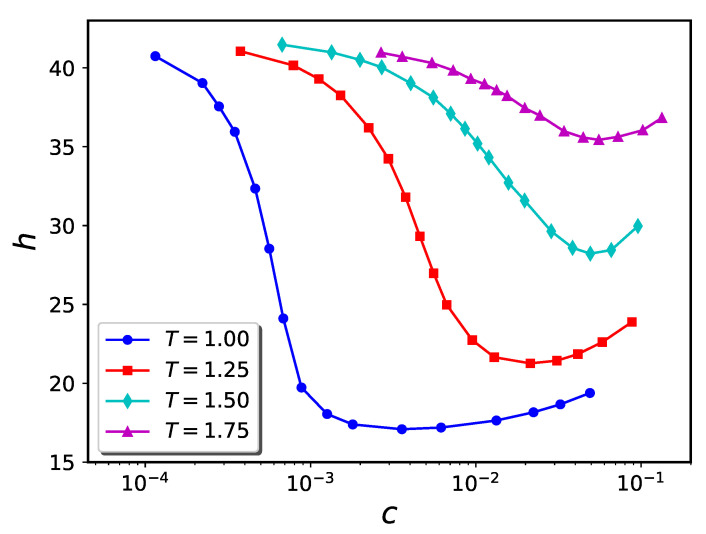
Similar to Figure 6 for $\epsilon_{b}=1.5$.

The computer simulation results for the particle trajectories were used to compute the brush height (*h*) vs. concentration of nanoparticles in the solution (*c*) for the attraction strengths $\epsilon_{b}=1.0$ and 1.5. These results are presented for $\sigma_{n}=1$ in Figure 6 and Figure 7, respectively. For the case of $\epsilon_{b}=1.0$, we observe that a rise in temperature from T=0.9 to 1.5 has a large impact on the extent to which the brush compresses as more nanoparticles are added to solution. For instance, the minimum value of the brush height changes from h=24.31 (for T=0.9) to h=36.87 (for T=1.2). Above T=1.5, the brush does not compress at any value of solution phase nanoparticle concentration. In this sense, the “collapse transition” is lost. Similar trends are observed when $\epsilon_{b}=1.5$.

From Figure 3 it is clear that the tethered polymer system is in the brush regime under conditions where N=100 and $\sigma_{g}=0.0625$ over a considerable range of nanoparticle concentrations and temperatures. It will remain a brush for longer polymer chain lengths, all other parameters being unchanged. The corresponding monomer density profile will change only by a scale factor, i.e., plotted vs. the reduced distance variable z/N, it will be a (nearly) universal function. (These statements are exact results of approximate mean field theories such as Milner-Witten-Cates [25,38,39] and Alexander-deGennes [40,41]. They are found to hold to a good approximation in MD simulations, provided that the polymer chains are long enough to drive the system of tethered polymers into a brush configuration.) Thus, simulations with the value of N=100 are sufficient to understand the brush morphology that will obtain for larger values of *N*.

**Figure 8:**
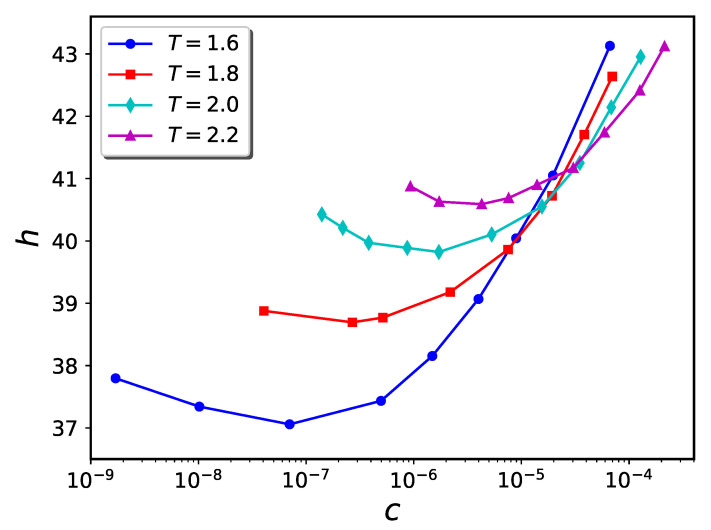
Temperature dependence of h−c diagram for the systems with nanoparticle diameter $\sigma_{n}=3$ and $\epsilon_{b}=1.25$ in flat geometry.

To study the effects of nanoparticle size, we present the *h* vs. *c* curves for $\sigma_{n}=3$ and $\epsilon_{b}=1.25$ in Figure 8. Increasing the nanoparticle size gives rise to two competing effects on the brush morphology: on one hand, it increases the surface area of the nanoparticle (by a factor of ${\left(\sigma_{n}^{\prime }/\sigma_{n}\right)}^{2}$). This in turn causes more monomer-nanoparticle binding energy per nanoparticle and hence favors a more collapsed brush. On the other hand, a nanoparticle absorbed into the brush partition occupies a much larger volume (by a factor of ${\left(\sigma_{n}^{\prime }/\sigma_{n}\right)}^{3}$), opposing brush collapse. The net result, as seen in the figure, is a slowed pace of brush collapse with increasing nanoparticle concentration. Another important point is that upon increasing the nanoparticle size $\sigma_{n}$, significant partitioning of nanoparticles into the brush occurs at much lower concentrations, which makes it computationally challenging to calculate the concentration *c*. (Hence we do not attempt to follow these *h* vs. *c* curves all the way to pure brush [*c* = 0] limit.) Apart from the above points concerning the nanoparticle size effects on the brush morphology, the trend in the temperature dependence of the h−c diagrams remains the same: the brush collapse transition disappears as temperature increases.

**Figure 9:**
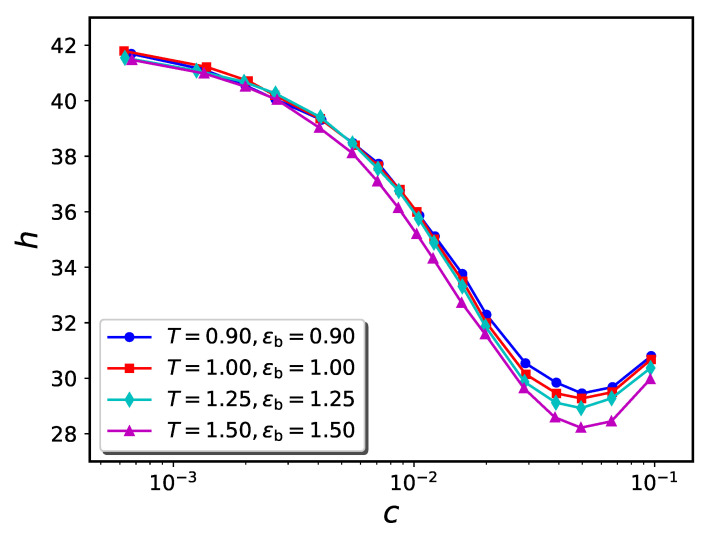
h−c diagrams for four different systems having the same value of $\epsilon_{b}/T=1.0$.

**Figure 10:**
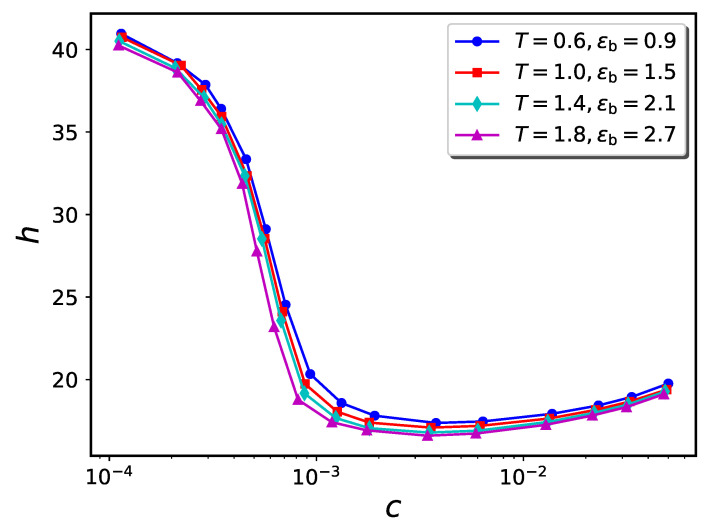
h−c diagrams for four different systems having the same value of $\epsilon_{b}/T=1.5$.

In Figure 9 and Figure 10, we compare *h* vs. *c* curves for 4 pairs of $\left(\epsilon_{b},T\right)$, all of which have the ratio $\epsilon_{b}/T=1.0$ and 1.5, respectively. As can be seen from the figures, the four curves are in close correspondence for both $\epsilon_{b}/T$ ratios. The dependence of equilibrium conformation properties solely on the ratio $\epsilon_{b}/T$ can be justified by considering the Boltzmann factor associated with each pair-wise interaction in the system. Properties such as the monomer or nanoparticle distributions depend directly on the total Boltzmann factor $\exp\{-U_{tot}\left({\vec{r}}_{1},\ldots ,{\vec{r}}_{n}\right)/k_{B}T\}$. Here $U_{tot}$ is the total potential energy of the system, which depends on the coordinates of each of the *n* particles in the system. $U_{tot}$ decomposes as the sum of all pair potentials between the various particles, so the total Boltzmann factor is a product of factors for each pairwise interaction.

Consider first a pure brush system. The pair-wise interactions here are of two types: a repulsive truncated LJ potential between each pair of particles, and an additional FENE spring potential between successive monomer chains on a given polymer chain. The repulsive truncated LJ potential is a good approximation to a hard core potential (infinite if the distance between the two particles is less than a particular value and zero if the distance is greater than this value). The FENE potential between adjacent monomers on a chain, in concert with the truncated LJ potential between these monomers, effectively restricts the distance between the two monomer centers to have a particular value. Both of these potentials can be described by functions which are either zero (in the region that the interparticle distance *r* can access) or infinity (in the region that the interparticle distance cannot access), leading to Boltzmann factors $\exp\left(-u\left(r\right)/k_{B}T\right)$, where u(r) is the appropriate interparticle potential, that have one of two values: 0 (in the forbidden region) or 1 (in the allowed region). Thus, the entire system potential $U_{tot}$ has the same property, i.e., $\exp\{-U_{tot}\left({\vec{r}}_{1},\ldots ,{\vec{r}}_{n}\right)/k_{B}T\}$ can be either 0 (if any pairwise potential term in $U_{tot}$ is infinite because the two particles involved are in a physically excluded configuration) or 1 (if no positional constraints are violated). In this case the system Boltzmann factor, which gives the relative probability for the system to have a given set of particle coordinates (and hence determines the monomer density) is independent of temperature *T*.

In a system comprised of both grafted polymer chains and nanoparticles that attract to polymer monomers, the nanoparticle-nanoparticle potential is purely repulsive, being represented by a truncated LJ potential as noted above. Hence the corresponding Boltzmann factor is essentially temperature independent. However, the nanoparticle-monomer pair potential has both a short range repulsive component (which can be approximated as a hard core repulsive potential) plus an “attractive tail”. The latter is a potential well having of finite depth $\epsilon_{b}$. Thus, the Boltzmann factor for each nanoparticle-monomer pair will be temperature dependent. Moreover, since the attractive tail potential $u_{well}$ is essentially proportional to $\epsilon_{b}$ (times a shape factor that is independent of $\epsilon_{b}$ and *T*), then $\exp(-u_{well}\left(r\right)/k_{B}T)$ will depend directly on the quantity $\epsilon_{b}/T$.

The statement that $\exp\{-U_{tot}/k_{B}T\}$ depends solely on the ratio $\epsilon_{b}/T$ (and hence is independent of *T* if $\epsilon_{b}=0$) is not exact, because the resemblance of the pair-potentials to the idealized forms noted above is only approximate. However, the resemblance is strong, so the approximation is good (see Figure 9 and Figure 10).

### 2.3. Brush plus Nanoparticles—Cylindrical Geometry

**Figure 11:**
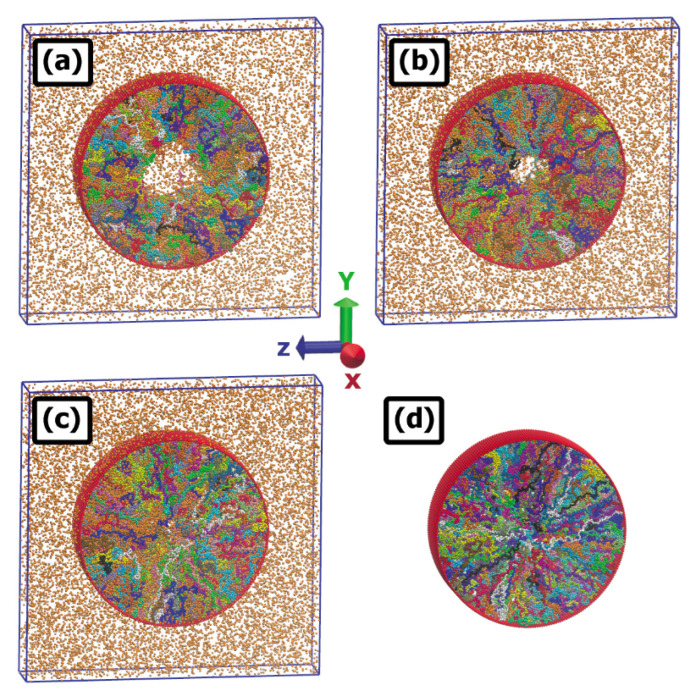
Snapshots of MD simulation cells for inside-grafted cylindrical nanopore. Results are shown for R=45, $\epsilon_{b}=1.25$ and temperatures T=0.9 (**a**), 1.0 (**b**), and 1.1 (**c**). Simulation setup consists of 400 polymer grafted chains together with 30000 nanoparticles. Panel (**d**) shows an MD snapshot from a pure brush (no nanoparticle) simulation run at T=1.0, included for comparison to systems (**a**–**c**).

**Figure 12:**
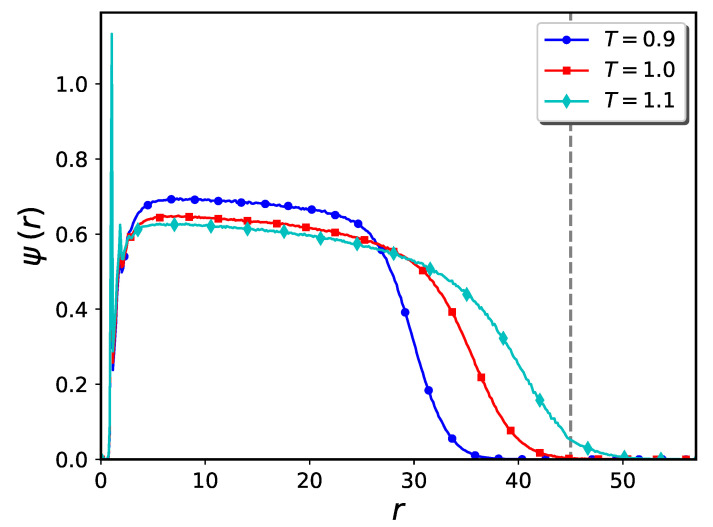
Monomer density profile of brushes at different temperatures corresponding to the snapshots shown in Figure 11a–c, computed via the projection method. The dash-line indicates the cylinder’s radius. The height of the pure brush is ca. 47.

**Figure 13:**
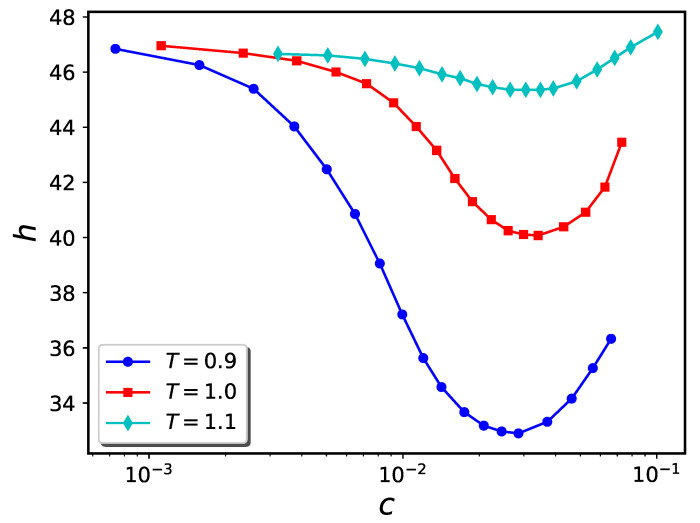
Temperature dependence of h−c diagram for inside-grafted cylindrical nanopore system containing 400 polymer chains, with nanoparticle diameter $\sigma_{n}=1$ and $\epsilon_{b}=1.0$. Brush heights are computed via the projection method.

Figure 2b shows that for the case of a pure cylindrical brush (no nanoparticles) with grafting density $\sigma_{g}=0.0625$, polymer chains of length N=100 are stretched at equilibrium such that their free ends extend slightly beyond the center of the pore (*R* = 45). They are thus poised to retract so as to open up a hole in the center of the pore if infiltrated by attractive nanoparticles. To study this effect, we carried out computer simulations in the cylindrical geometry in the presence of attractive nanoparticles. Results of these simulations are presented in Figure 11, Figure 12 and Figure 13. Figure 11 shows snapshots of the simulation box at 3 different temperatures. In our simulations, we used 400 polymer chains randomly grafted inside a cylinder with radius R=45 and 30,000 nanoparticles having diameter $\sigma_{n}=1$. The monomer-nanoparticle binding strength parameter was $\epsilon_{b}=1.0$. The cylinder axis was fixed along the *x* direction. The cylinder wall was made of atoms which were permeable to the nanoparticles (see Ref. [4] for details). In Figure 11, at the lowest temperature (panel a) there is a relatively large hole in the middle of the cylinder. Upon increasing the temperature (panel b) the hole becomes smaller, and upon further temperature increase (panel c) it disappears. In panel (d) of this figure a snapshot of the corresponding pure brush (nanoparticle free) system at temperature T=1.0 is presented for comparison. As shown above, the brush morphology varies little with temperature in the absence of nanoparticles. Thus, as was true in the flat brush case, it is apparent that having attractive nanoparticles in the system is required for the brush height to change significantly when the system temperature is altered.

To compute the density profile of polymer brushes in cylindrical systems, we may use either radial or projection methods (see Ref. [4] for a detailed description of the projection method). If the polymer chains extend beyond the center of cylinder, for a meaningful determination of the brush height we are limited to using the projection method. We used this method to compute the density profile of the systems shown in Figure 11a–c. The results are presented in Figure 12. The value of the cylinder radius is indicated in the figure by the dashed vertical line. At the lowest temperature, T=0.9, the density profile decays to zero at values of *r* which are far less than the cylinder radius, indicating the presence of a large hole in the middle of cylinder. At T=1.0, the brush tip touches the cylinder’s center, which means the hole is much smaller. Finally, at the highest temperature, T=1.1, the density profile extends beyond the cylinder’s center and thus the cylinder is closed (although the monomer density close to the center is small).

To compute the density of nanoparticles in the solution, we count the number of nanoparticles outside the cylinder and divide it by the volume of the whole simulation box minus the volume of the cylinder. The resultant h−c diagrams are shown in Figure 13. Unlike the pure brush analog (see Figure 2, panel b) the brush height is highly sensitivity to temperature changes.

## 3. Discussion

In this paper, we have studied the morphology of a polymer brush in a good solvent when attractive nanoparticles are added, focusing on the effect of temperature on the density distribution of monomers and nanoparticles in the brush. By performing coarse-grained MD simulations, we found that a system with no nanoparticles (pure brush) has only weak temperature dependence, as expected when it is immersed in a good solvent. However, when nanoparticles that attract to the monomers of the polymer chain are present, the brush morphology exhibited significant temperature dependence. Moreover, the effect of temperature on brush morphology in the presence of nanoparticles depended, to a good approximation, solely on the ratio $\epsilon_{b}/T$. These findings have practical importance for the design of smart polymer devices that rely on external control of the polymer brush morphology (e.g., extent of swelling or compression). Our results make clear that temperature provides another “tuning knob” for these processes. It induces the same types of change as does modification of the binding strength $\epsilon_{b}$ (for example, increasing $\epsilon_{b}$ at fixed *T* has the same effect as decreasing *T* at fixed $\epsilon_{b}$). However, changing temperature is easier experimentally than changing $\epsilon_{b}$, as the latter requires chemical synthesis and is not easily reversible the way temperature changes are.

It has previously been shown that polymers whose morphologies are thermo-responsive due to the existence of enthalpic solvent-monomer interactions hold promise for utilization in nanodevices [42,43]. The present work shows that a similar kind of thermo-responsivity can be obtained using nanoparticle-infiltrated polymer brushes. The ability to easily and reversibly adjust two experimental parameters, namely temperature and nanoparticle concentration, extends the degree of control over brush morphology relative to the situation where only one external parameter (e.g., nanoparticle concentration alone) can be tuned. As can be seen from Figure 6, Figure 7 and Figure 13, the value of the nanoparticle concentration determines how the brush extension varies with system temperature. At an appropriate nanoparticle concentration, significant contraction or expansion of the brush can be attained with modest temperature changes. For example, (cf. Figure 11), a polymer brush coated cylindrical nanovalve can be opened or closed with modest temperature changes. 

## 4. Methods and Materials

We carried out equilibrium MD simulations in the constant NVT ensemble to study the structural properties of a system of grafted polymer chains on flat and cylindrical surfaces in the absence of and in the presence of additional mobile nanoparticles. For the polymer chains, we adopt the interaction potential proposed by Kremer and Grest [44] in which all monomers are spherical, having mass *m* and diameter $\sigma_{m}$, with a non-bonded interaction potential between all monomers modeled via a truncated and shifted Lennard-Jones (LJ) potential

(1) $$U_{mm}\left(r\right)=\left\{\begin{array}{cc} 4\epsilon \left[{\left(\sigma_{m}/r\right)}^{12}-{\left(\sigma_{m}/r\right)}^{6}\right]+\epsilon , & r<r_{c,m}.\\ 0 & r>r_{c,m} \end{array}\right.$$

The potential energy function $U_{mm}$ in Equation (1) has no well, and $r_{c,m}=2^{1/6}\sigma_{m}$ is the cutoff distance. Each pair of adjacent monomers in a given polymer chain interacts via the finitely extensible nonlinear elastic (FENE) potential defined by

(2) $$U_{\mathrm{FENE}}\left(r\right)=-\frac{1}{2}kr_{0}^{2}\ln\left[1-{\left(\frac{r}{r_{0}}\right)}^{2}\right],$$

where $k=30\epsilon /\sigma_{m}^{2}$ is the spring constant, and $r_{0}=1.5\sigma_{m}$ is the bond’s maximum extension.

The nanoparticles are taken to be spherical with a mass identical to the polymer beads. (We are interested here in equilibrium properties that depend only on the configuration integral [45]. These properties are independent of particle masses. Thus, the values of the particle masses are arbitrary for present purposes.) Two nanoparticle diameters are considered: $\sigma_{n}=\sigma_{m}$ and $3\sigma_{m}$. The interaction between nanoparticles is purely repulsive (analogous to Equation (1), with $\sigma_{m}\to \sigma_{n}$). The interaction between nanoparticles and polymer beads, on the other hand, includes an attractive tail, as described by the modified Lennard-Jones potential

(3) $$U_{nm}\left(r\right)=\left\{\begin{array}{cc} 4\epsilon_{b}\left[{\left(\sigma_{m}/\left(r-\Delta \right)\right)}^{12}-{\left(\sigma_{m}/\left(r-\Delta \right)\right)}^{6}\right], & r<r_{cut},\\ 0 & r>r_{cut} \end{array}\right.$$

where $\Delta =(\sigma_{n}-\sigma_{m})/2$, and $r_{cut}=3\sigma_{m}$ is the potential cutoff distance. This potential possesses a well of depth $\epsilon_{b}$ with its minimum located at approximately $r=(\sigma_{n}+\sigma_{m})/2$.

We utilized the LAMMPS molecular dynamics simulation software [46] for all the simulations in this work and applied the VMD program [47] for visualization purposes. The polymer chains are grafted randomly at one end either to the surface of a rectangular simulation box (flat geometry) or on the internal surface of a cylinder (inside-grafted cylinder geometry). In the flat (planar) geometry the simulation box is bounded by two walls in the *x*-*y* plane at the bottom and top surfaces in the *z* direction, and periodic boundary conditions are applied in the *x* and *y* directions. For the cylinder, periodic boundary conditions are used in all three Cartesian directions, including the direction of the cylinder axis. For the polymer brush-nanoparticle mixture, the cylinder is immersed inside a large simulation cell with the particles comprising the cylinder wall being permeable to the nanoparticles. The density profile of the monomers is computed by using snapshots extracted from the simulation runs. For the plane-grafted systems, the density profiles are normalized via the relation

(4) $$\int_{0}^{L_{z}}\psi \left(z\right)dz=\sigma_{g}N,$$

where $\sigma_{g}$ is the polymer grafting density, *N* is the number of monomers per chain, and $L_{z}$ is the box length in the *z* direction. With this normalization, ψ(z) is identified as the concentration (or monomer density) locally at point *z*. For cylindrical systems, we obtain the monomer density profile ψ(r) by using the normalization relation [4]

(5) $$2\pi L\int_{0}^{R}\left(R-r\right)\psi \left(r\right)dr=Nn_{ch},$$

where *L* is the cylinder length, *R* is the cylinder radius, and $n_{ch}$ is the number of polymer chains. The radial coordinate of the cylinder, *r*, is measured here as the distance from the grafting wall. Direct computation of the local monomer number density at point $\vec{r}$, which gives rise to the function ψ(r) introduced above (designated as the radial method), is not able to resolve the extension of the polymer chains if they stretch beyond the cylinder radius R. In this case, there is another method of accounting for the monomer distribution, to be termed the projection method, which provides a sensible measure of the brush height when the latter is greater than R, as detailed in Ref. [4].

Upon adding nanoparticles to the simulation system, two phases are formed: a polymer brush layer with nanoparticles in it and a solution phase containing only nanoparticles. We are interested in computing the brush height (*h*), and the concentration of nanoparticles in the solution (*c*). Because the unit of length employed in the simulations is the monomer diameter $\sigma_{m}$, the volume fraction occupied by the monomers at point *z* is essentially equal to their concentration there. For flat systems, in order to calculate the brush height from the computed density profile, we use the relation

(6) $$\frac{\int_{0}^{h}\psi \left(z\right)dz}{\int_{0}^{L_{z}}\psi \left(z\right)dz}=0.995.$$

As in our previous work [3,4,5], we used the value 0.995 in the above relation by calibrating our analysis of the simulation results for a pure brush with those reported by Murat and Grest [48]. For cylindrical systems, we use a similar equation to compute the brush height. The concentration *c* is computed by counting the number of nanoparticles in the solution divided by the volume of the solution partition.

A total of 100 and 400 chains were used in our simulations of flat and cylindrical systems, respectively. Each polymer chain consisted of 100 beads and the chains were grafted randomly at one end. We picked the cylinder radius $R=45\sigma_{m}$ to be consistent with our previous studies [3,4]. The initial configuration in all of our simulations consists of grafted polymer chains that are fully stretched out. This prevents the system from becoming trapped in a metastable (non-equilibrium) conformation when the MD simulation commences. For each state point, we performed five simulation runs and used the standard deviation of the results to quantify the fluctuations in the calculated properties. These standard deviations turned out to be modest, on the size of the symbols used to plot each data point, and thus are suppressed in the plots of MD simulation data presented below.

We used the velocity-Verlet algorithm to solve Newton’s equations of motion numerically with time step of δt=0.005τ, where $\tau =\sigma {\left(m/\epsilon \right)}^{1/2}$. The simulations were carried out in constant NVT ensemble and a Langevin thermostat was applied to control the temperature with a damping constant of Γ=1/τ. The equilibration and production runs were performed for $4\times 10^{6}$ and $10^{6}$ time steps, respectively.

## Data Availability

The data presented in this study are available on request from the corresponding author.
